# Quantitative parameter mapping of contrast agent concentration and relaxivity and brain tumor extracellular pH

**DOI:** 10.1038/s41598-022-05711-z

**Published:** 2022-02-09

**Authors:** Yuki Matsumoto, Masafumi Harada, Yuki Kanazawa, Yo Taniguchi, Masaharu Ono, Yoshitaka Bito

**Affiliations:** 1 Graduate School of Biomedical Sciences, Tokushima University, Tokushima City, 770-8503 Japan; 2 FUJIFILM Healthcare Corporation, Tokyo, 107-0052 Japan

**Keywords:** Diagnostic markers, Cancer imaging

## Abstract

In clinical magnetic resonance imaging, gadolinium-based contrast agents are commonly used for detecting brain tumors and evaluating the extent of malignancy. We present a new method to evaluate relaxivity (r1) and contrast agent concentration separately in contrast-enhanced lesions using quantitative parameter mapping (QPM). Furthermore, we also aimed to estimate the extracellular pH (pHe) of tumor lesions. We demonstrated that it is possible to evaluate pathophysiological tumor changes due to therapeutic efficacy by measuring r1 in contrast-enhanced lesions. In this study, the primary brain tumor group showed significantly higher r1 values than other brain disease groups (*P* < 0.001). Moreover, mean pHe value showed a trend for tumor malignancy having a lower pHe value and primary brain tumor having a significantly lower pHe than other brain diseases (P < 0.001). Our results might suggest that QPM can separately quantify r1 and CA concentration in brain tumors and that pHe brain tumor mapping could serve as a tumor biomarker. In conclusion, our method has potential clinical applications for assessing the treatment effects.

## Introduction

The effect of contrast enhancement by a gadolinium (Gd)-based contrast agent (CA) depends on both its concentration and longitudinal relaxivity (r1) in lesions. It is well known that the r1 of a Gd-based CA is affected by the rate of exchange interaction of water protons, which is influenced by factors around the lesion area, such as pH, temperature, and diffusivity^1–3^. Therefore, the r1 of a CA may reflect the biological environment of the lesion and act as an index of the lesion microenvironment. The pharmacokinetic parameter of Gd-based CAs is spin–lattice relaxation time constant (*T*_1_)-shortening because of leakage to extracellular tissues from cerebral vessels owing to blood–brain barrier destruction, which allows the CA to leak out into the extracellular space^4^. The mechanism of the shortened-T1 is mainly due to dipole–dipole interactions of coordinated water molecule with the Gd3+ complex^5,6^. Furthermore, the acidic environment caused by the anabolic metabolism in the extracellular space can act as a physical source for Gd3+ relaxation changes, as the hydration state (q) of molecular CAs plays a role in modulating relaxation changes under acidic conditions.

The concentration of CA and CA’s r1 should be independently measured because CA concentration in a lesion depends on several factors including pathophysiological conditions. Quantitative parameter mapping (QPM) is a recently proposed method among synthetic magnetic resonance imaging (MRI) techniques^7^; it uses three-dimensional (3D) RF-spoiled gradient-echo pulse sequences with multiple repetition times, echo times, and flip angle values. The intensity function of the rapid imaging of RF-spoiled gradient echo is then formulated using computer simulation based on Bloch equations. This allows simultaneously acquisition of multiple MR parameter maps, such as T1 and T2 maps, by applying a pulse sequence in which the intensity depends on these MR parameters. One of the important advantages of QPM is that both relaxation and quantitative susceptibility mapping (QSM) can be independently obtained. We considered that QPM before and after CA injection would allow separate quantification of both r1 and CA concentration using two parameters derived from subtracted longitudinal relaxation rate (R1) and QSMs.

Several studies have shown pH dependency of relaxivity measurements using pH-responsive CAs^8–11^. Although it is possible to assess pH changes by measuring relaxation rates, few studies have demonstrated the relationship between relaxation rate and pH of commercially available Gd-based CAs^8,9^.

This study aimed to separately assess the effect of CA’s r1 value and concentration to evaluate the possibility of a new index for r1 and measure extracellular pH (pHe) values to obtain pathophysiological information on brain tumors. Therefore, we performed a phantom experiment to investigate the r1 value of a commercially available Gd-based CA, Gd-BTDO3A, and evaluated the relationship between r1 and pH to develop a pH calibration curve at each r1 value in Gd-BTDO3A using a non-linear function, as previously reported^9^.

## Methods

### Subjects and data acquisition

All clinical investigations were conducted according to the principles expressed in the Declaration of Helsinki. To verify that the pHe map can be measured both before and after CA injection, patients with brain tumor (radiation necrosis: three lesions in three patients, brain metastasis: twelve lesions in four patients, primary brain tumor: two lesions in two patients), all of whom provided informed consent, underwent MRI (Table 1). This study was approved by our institutional review board (Tokushima University Hospital), and all MRI data acquired using a 3-T system (FUJIFILM Healthcare Corporation, Japan) with a 32-element-phased array receive coil. QPM employs 3D partially radio frequency (RF)-spoiled steady state gradient-echo (3D-pRSSG) methods with multiple repetition time, echo-time, and flip angle values. To achieve adequate T1 and T2 relaxation times, imaging parameters are optimized using the law of error propagation with target relaxation times at 3 T. In addition, the first RF excitations (up to 50 cycles) were skipped to reach steady state. Imaging parameters were as follows: echo times, 4.5–36.8 ms; repetition times, 10–41.3 ms; and flip angles, 10°–40°. Image resolution was 0.94 × 0.94 × 2 mm with a 240 × 240 mm^2^ FOV. Parallel imaging was used to reduce overall scan time, and the acceleration factor was 1.9 × 1.9 (RL × AP). CA amounted to 0.1 mmol/kg of body weight and was injected into the patient’s vascular system. Whole-brain acquisition was then performed using the same imaging parameters as pre-injection. Note that the whole brain was scanned ≥ 1 min after CA injection. Total scan time was under 19 min, both before and after CA injection. In addition, a susceptibility map was used, which was reconstructed using the QSM algorithm with multi-echo images, as part of the QPM dataset. Multi echoes were used (five echoes, 4.5–36.8 ms, ΔTE = 6.9 ms), with repetition time and flip angle of 40 ms and 10°; other imaging parameters were the same as with the QPM dataset.

**Table 1:** Patient data.

Age (years)	Sex	Clinical diagnosis	Group
79	F	Radiation necrosis #1	Radiation necrosis
61	F	Radiation necrosis #2	Radiation necrosis
72	F	Radiation necrosis #3	Radiation necrosis
68	M	Metastasis #1	Metastasis
72	F	Metastasis #2	Metastasis
74	M	Metastasis #3	Metastasis
47	F	Metastasis #4	Metastasis
49	F	Anaplastic astrocytoma	Primary brain tumor
76	F	Glioblastoma	Primary brain tumor

### Phantom experiment for pH calibration

r1 is generally defined as the slope of the resulting fit from a linear regression of the measured R1 (i.e., 1/T1) of the tissue and CA concentration^12,13^ as follows:

1a $${R1}_{post}= {R1}_{pre}+r1 \times CA$$

1b $$\therefore r1= \frac{{R1}_{post}- {R1}_{pre}}{CA}$$

where R1_pre_ and R1_post_ denote the R1 values of the tissue before and after CA injection, respectively. CA denotes the CA concentration. The relaxivity r1 can thus be estimated by measuring R1_pre_, R1_post_, and CA, as shown in Eq. (1b). Here, the measured relaxivity value is different in acidic pH; thus, the empirical relationship between those values must be investigated to calculate the pHe. To validate r1 depending on the pHe, a phantom experiment was then performed using a pH buffer solution, and r1 at each pH acidity was measured. We prepared various pH solution samples of known acidity (pH range of 6.0–7.8), including the CA Gd-BTDO3A (Gadovist, Bayer HealthCare) at different concentrations (0.1, 0.2, 0.4 and 0.5 mmol). The pH of these samples was achieved by mixing sodium dihydrogen phosphate dihydrate (0.2 mmol) and di-sodium hydrogen phosphate 7-hydrate (0.2 mmol) solutions. Additionally, real pH solutions were measured with a calibrated HI 2020-01 pH meter (HANNA instruments, USA). Then, the sample containers were placed in the MR exam table so that the axes of the sample containers were perpendicular to the main field, and a 3 T MRI system (FUJIFILM Healthcare Corporation, Japan) used at room temperature (20 °C) and body temperature (37 °C). To measure the T1, a single slice of a coronal scan was obtained using a fast spin-echo based inversion recovery (FSE-IR) with the following parameters. Inversion time (TI) was performed at 40, 100, 160, 240, 300, 400, 500, 600, 800, 1000, 1200, 1500, and 2000 ms. Then, 7000 ms of TR was chosen to assure TR ≫ T1. The shortest possible effective echo-time value (eTEs) was used: 78.0 ms. The other imaging parameters were as follows: field of view (FOV), 288 mm; imaging matrices and reconstruction matrices, 288 × 201.6 mm^2^; and slice thickness, 6 mm.

### Determination of the pH-relaxivity curve

Here, we describe how the pH-relaxivity calibration curve can be calculated from T1 maps. Non-linear least square fitting was first performed to yield longitudinal relaxation times T1 using the following equation^13^:

2 $${S}_{TI}=abs\left[M0\times (1-(1-k)\times \mathrm{exp}(\frac{-TI}{T1}))\right],$$

where SI defines the MR signal, and the subscript TI refers to each variable obtained at TI. M0 and T1 define equilibrium magnetization and longitudinal relaxation times, respectively. The mean R1 value of each container was determined by drawing a region-of-interest (ROI), which was an equally sized square shape (64 pixels). After a linear regression analysis based on Eq. (1b) was performed, r1 values were individually calculated at each pH. In this study, the pH-sensitive range was chosen as previously reported^14^ because it exhibits unique characteristics depending on the CA’s chemical structure. The pH can then be calculated using least square fitting of Hill-modified Henderson–Hasselbalch equation:

3 $${pH=pKa-{log}_{10}\left[\frac{r1-{r1}_{base}}{{r1}_{acid}-r1}\right]}^{n}$$

where r1 defines relaxivity, and pKa, r1_base_, r1_acid_, and n must be determined according to the phantom experiment.

### Calculation of relaxivity values

**Figure 1:**
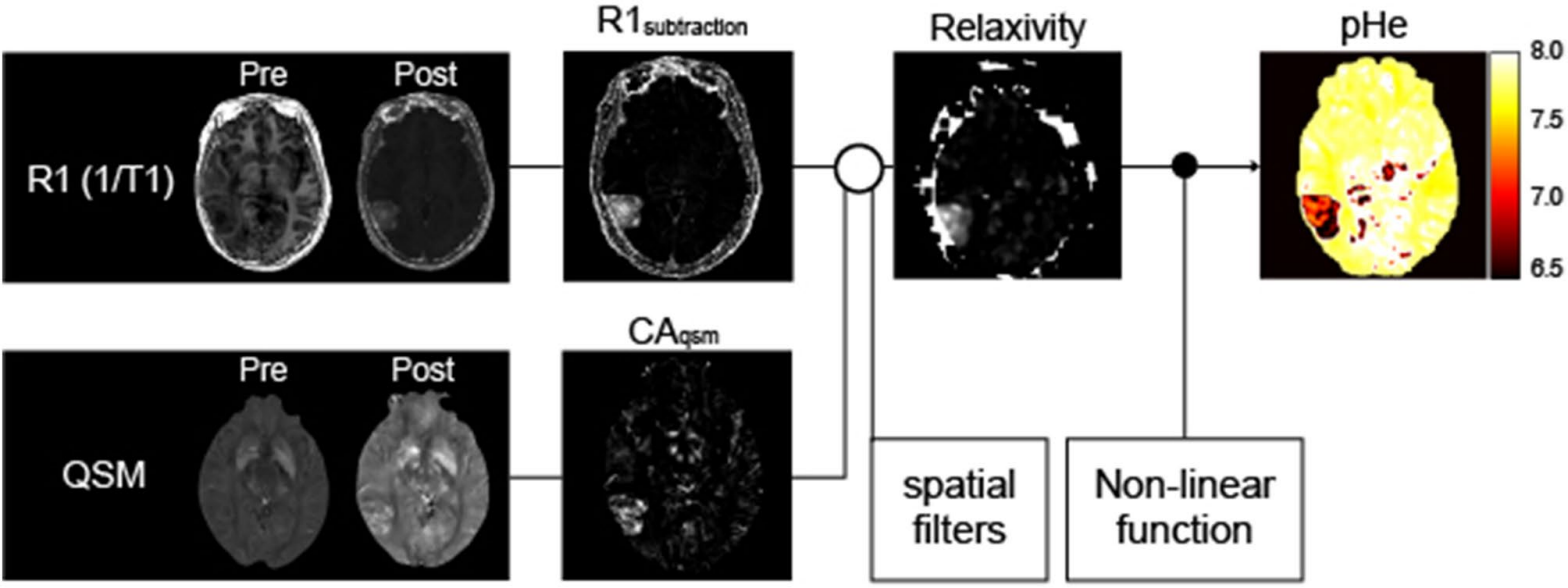
Scheme of the procedure for extracellular pH (pHe) calculation. Relaxivity (r1) and susceptibility maps were calculated from both quantitative parameter mapping (QPM) datasets before and after contrast agent (CA) injection. The subtraction (*R*_1substraction_) map and the CA concentration map (CA_qsm_) were then calculated from these R1 and susceptibility maps, respectively (see Eqs. 4 and 5). The relaxivity map was calculated by dividing the *R*_1substraction_ by CA_qsm_ each voxel. The pHe was finally estimated by applying a pH calibration curve at body temperature (37 °C).

An overview of data processing is provided in Fig. 1. There are several processing steps to estimate r1 before and after CA injection of the contrast agent. First, R1 and susceptibility maps were calculated from both QPM datasets before and after CA injection. The subtraction (*R*_1substraction_) map and the CA concentration map (CA_qsm_) were then calculated as follows:

4 $${R1}_{subtraction}={R1}_{post}-{R1}_{pre},$$

5 $${CA}_{qsm}=\frac{{\chi }_{post}-{\chi }_{pre}}{{\chi }_{gd}}\times {Mol}_{Gd},$$

where ${\chi }_{pre}$ and ${\chi }_{post}$ define susceptibility values before and after CA injection, respectively, and ${\chi }_{Gd}$ defines CA’s susceptibility^15^. In addition, ${Mol}_{Gd}$ defines a CA’s molar concentration. In this study, multiple dipole-inversion combination with k-space segmentation (MUDICK) and 326 ppm were used for estimating ${{\chi }_{pre}, \chi }_{post}$ and ${\chi }_{Gd}$, respectively^16,17^. The relaxivity *r*_1_ was subsequently estimated with Eq. (1b) by measuring *R*_1 subtraction_ and CA_qsm_. Here, a linear regression analysis was performed to confirm linearity between the CA_qsm_ and the *R*_1subtraction_ in the brain tumor. To remove division artifacts, a 3D median filter and Gaussian filter (standard deviation of 1) were applied to *r*_1_. Finally, the pHe map was calculated based on the resulting *r*_1_ map and the non-linear function derived from the phantom experiment. Of note, the pHe value can only be measured in contrast-enhanced areas caused by the disrupted blood–brain barrier. It is therefore impossible to compare the pHe value on the tumor region and that of normal tissue, such as normal appearing white matter.

### Statistical analysis

After administering the contrast, ROIs were drawn on the R1 map and applied to the quantitative maps of R1 _subtraction_, CA_qsm_, r1 and pHe. As the mean pH difference based on tumor malignancy would indicate the clinical potential of brain tumor pHe, a one-way analysis of variance (ANOVA) test was used to observe whether changes in the mean pHe and r1 were dependent on malignancy. To estimate the pH value of the blood, ROIs were also drawn on the superior sagittal sinus using ~ 10 slices on the R1_subtraction_ each patient and applied to the pHe map.

## Results

### Phantom experiment

**Figure 2:**
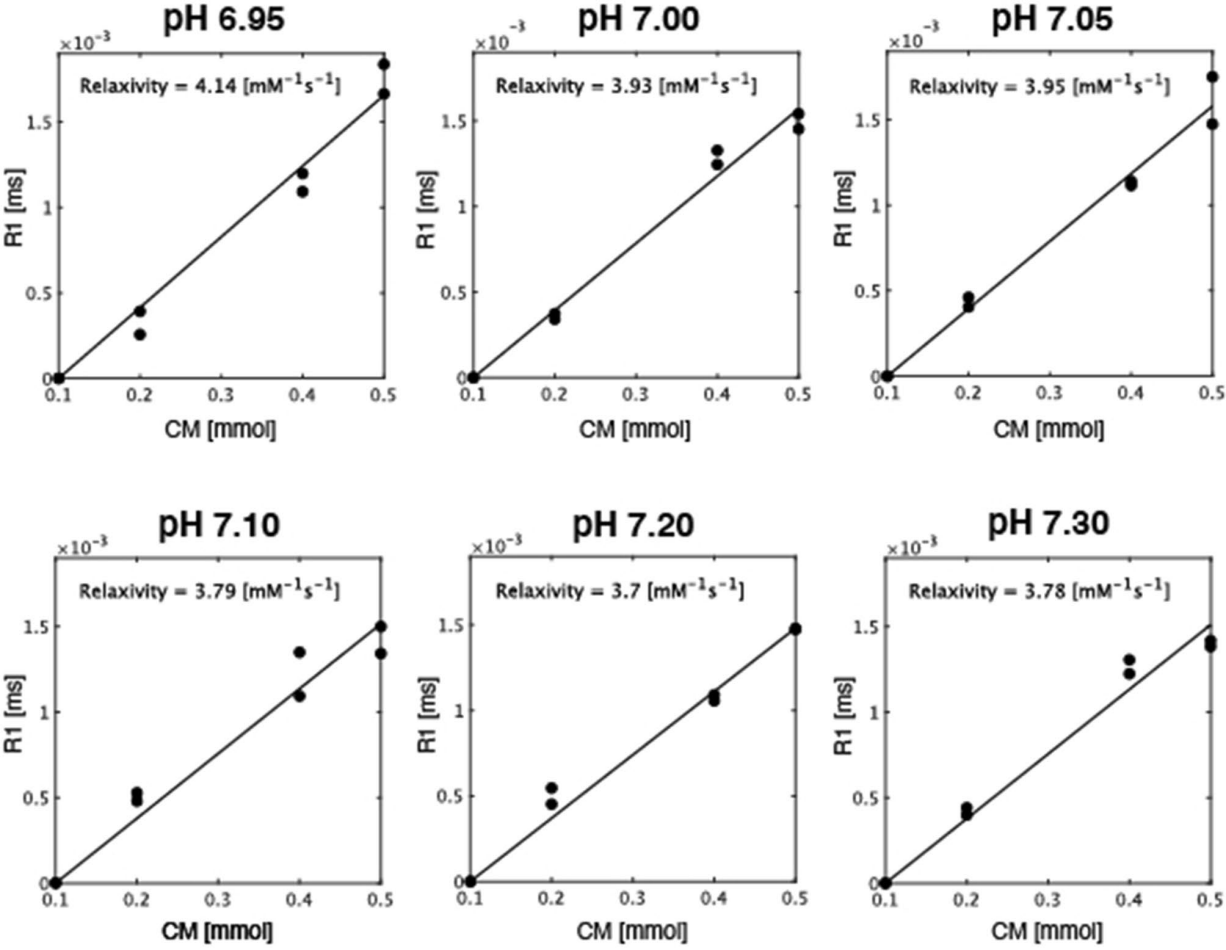
Determination of Gd-BTDO3A relaxivity at 3.0 T MRI. Relaxation rates (1/T1) were plotted against the concentration of four Gd-BTDO3A concentrations (0.1, 0.2, 0.4 and 0.5 mmol) at body temperature (37 °C), respectively. The relaxivity value was then determined from the slope of the linear regression of each pH solution.

**Figure 3:**
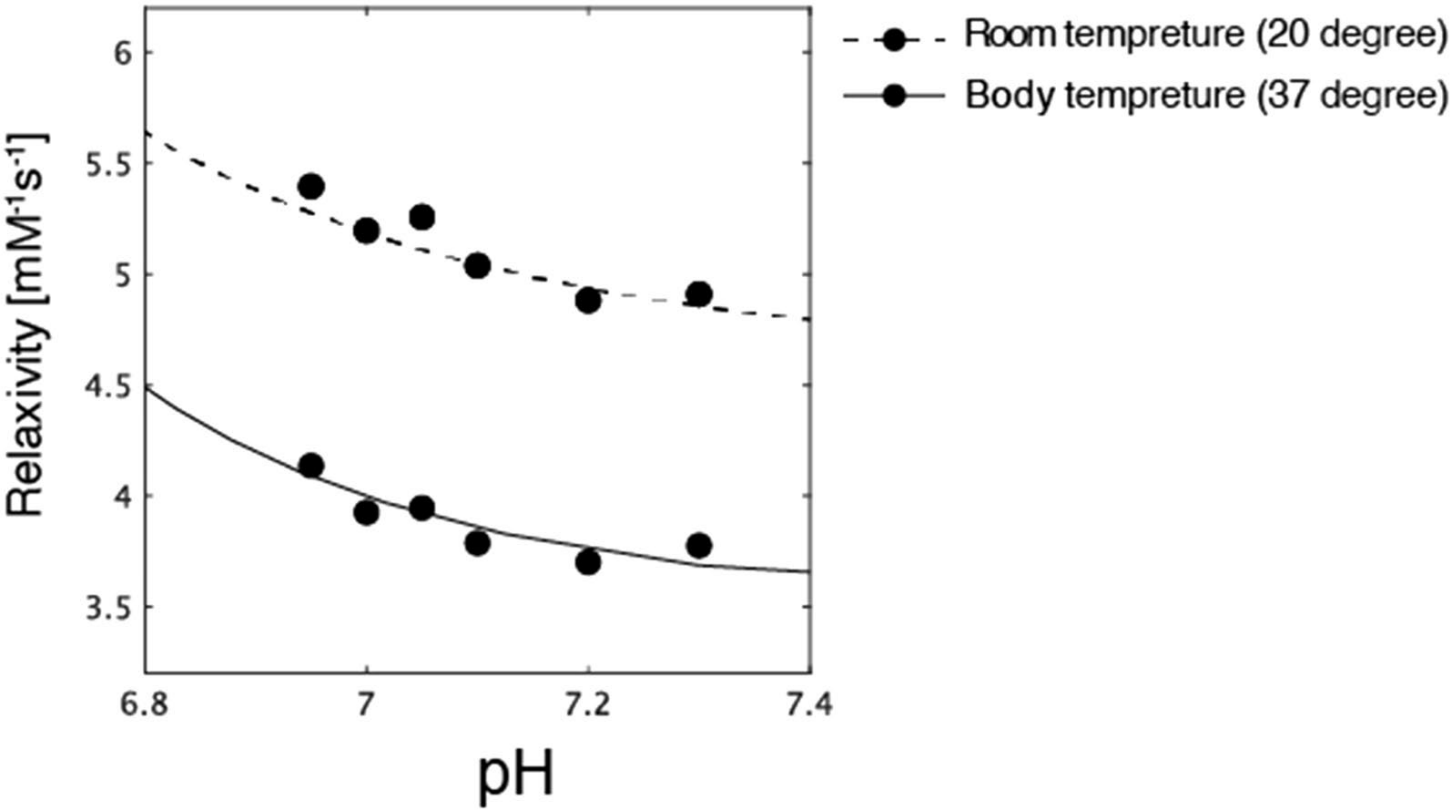
Determination of the pHe-relaxivity calibration curve for Gd-BTDO3A. These measurements were performed at room temperature (20 °C) and body temperature (37 °C) on a clinical 3.0 T MRI scanner.

Figure 2 shows the relaxivity determination of Gd-BTDO3A at 3.0 T MRI. Relaxation rates (1/T1) were plotted against the concentration of four Gd-BTDO3A examples at body temperature (37 °C). Figure 3 shows r1 depending on a pH range at room temperature and body temperature, respectively. The pH calibration curve of body temperature was defined as the pH-sensitive ranges after confirming the relaxivity behavior from Fig. 2. In this study, a range of 6.95–7.3 was considered as a pH-sensitive range, and a non-linear regression analysis performed. The fitted result led to the following values: kPa = 6.70, r1_base_ = 3.50, r1_acid_ = 6.50, and n = 1.34.

### Brain tumor

**Figure 4:**
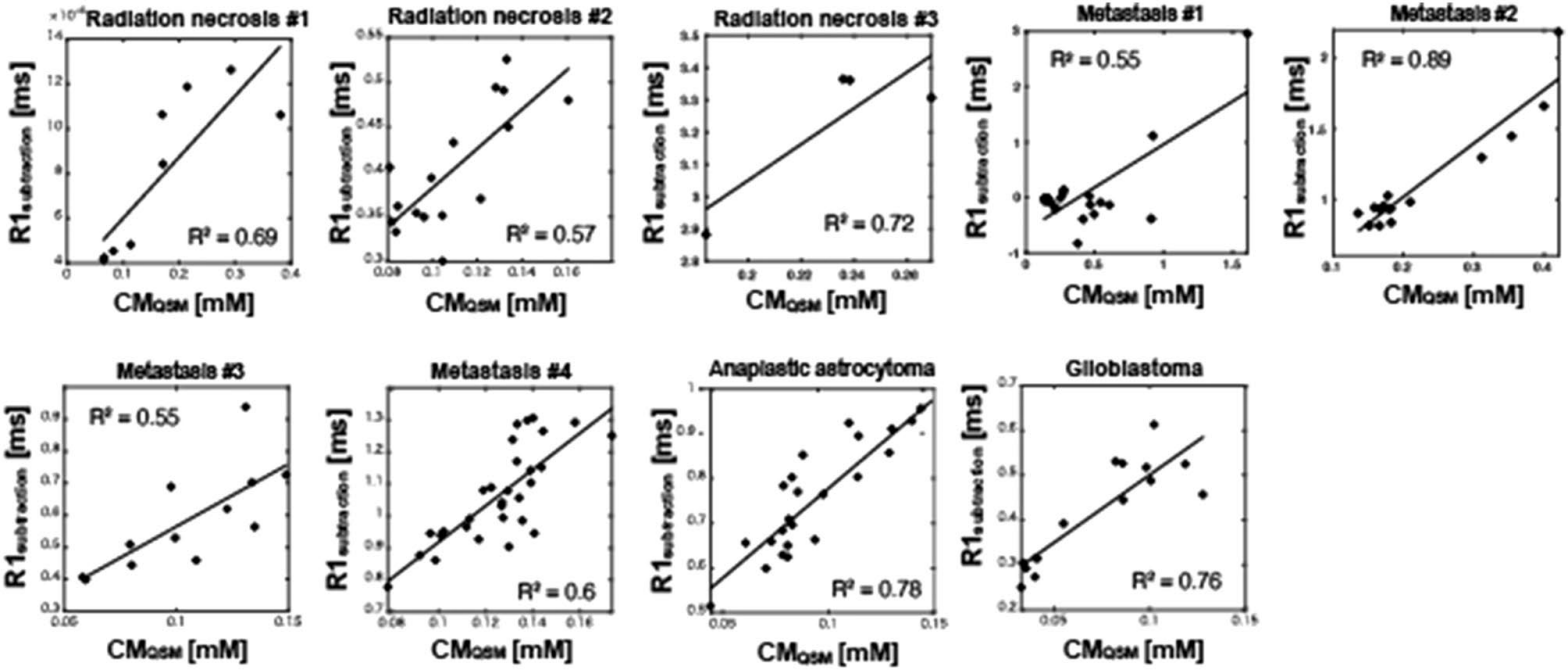
Relationship between *R1*_subtraction_ and CA_qsm_ for each brain disease. Mean *R1*_subtraction_ values were plotted against mean CA_qsm_ values on tumor lesions. The tumor lesions were defined by drawing region-of-interest (ROI) on the R1 map after contrast agent injection.

**Figure 5:**
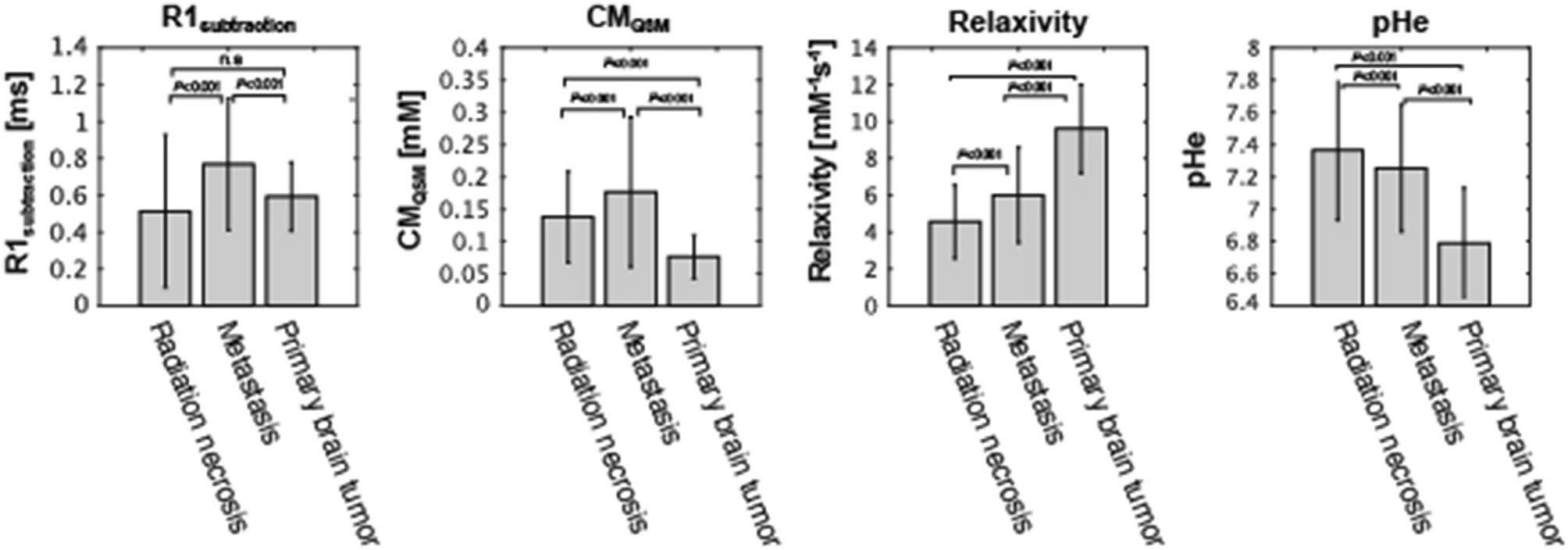
Mean R1 subtraction, CA_qsm_, relaxivity, and pHe values among patient groups. The mean pHe value showed a trend for tumor malignancy having a lower pHe value and primary brain tumors having a significantly lower pHe than other brain diseases (*P* < 0.001).

The comparisons demonstrating a strong correlation between CA_qsm_ and R1 subtraction maps of all tumor lesions are shown in Fig. 4 (R^2^ ≥ 0.55). In this study, the relationship between CA_qsm_ and *R*_1 subtraction_ was individually plotted because of the mean relaxivity value, defined as the slope of the resulting fit from the linear regression. The mean brain tumor r1 changed with the anabolic metabolism in the extracellular space, indicating that CA’s r1 can be independently measured by R_1 subtraction_ and CA_qsm_. The mean values of *R*_1 subtraction_, CA_qsm_, relaxivity, and pHe are shown in Fig. 5. Table 2 also shows the mean values of *R*_1 subtraction_, CA_qsm_, relaxivity, and pHe for each patient.

**Table 2:** Mean CA, relaxivity, and pHe values each of the patients.

Age (years)	Sex	Clinical diagnosis	CA (mmol)	Relaxivity (mM^−1^ s^−1^)	pHe
79	F	Radiation necrosis #1	0.16 ± 0.22	0.01 ± 0.00	7.81 ± 0.00
61	F	Radiation necrosis #2	0.13 ± 0.12	1.69 ± 2.53	7.34 ± 0.90
72	F	Radiation necrosis #3	0.24 ± 0.32	4.61 ± 3.39	7.12 ± 0.63
68	M	Metastasis #1	0.23 ± 0.32	11.32 ± 6.64	7.75 ± 0.64
72	F	Metastasis #2	0.11 ± 0.13	5.23 ± 2.09	6.97 ± 0.71
74	M	Metastasis #3	0.23 ± 0.19	5.91 ± 2.41	6.79 ± 0.67
47	F	Metastasis #4	0.13 ± 0.12	9.4 ± 4.50	6.95 ± 0.66
49	F	Anaplastic astrocytoma	0.08 ± 0.07	9.54 ± 4.74	6.79 ± 0.81
76	F	Glioblastoma	0.05 ± 0.05	9.82 ± 5.01	6.79 ± 0.99

**Figure 6:**
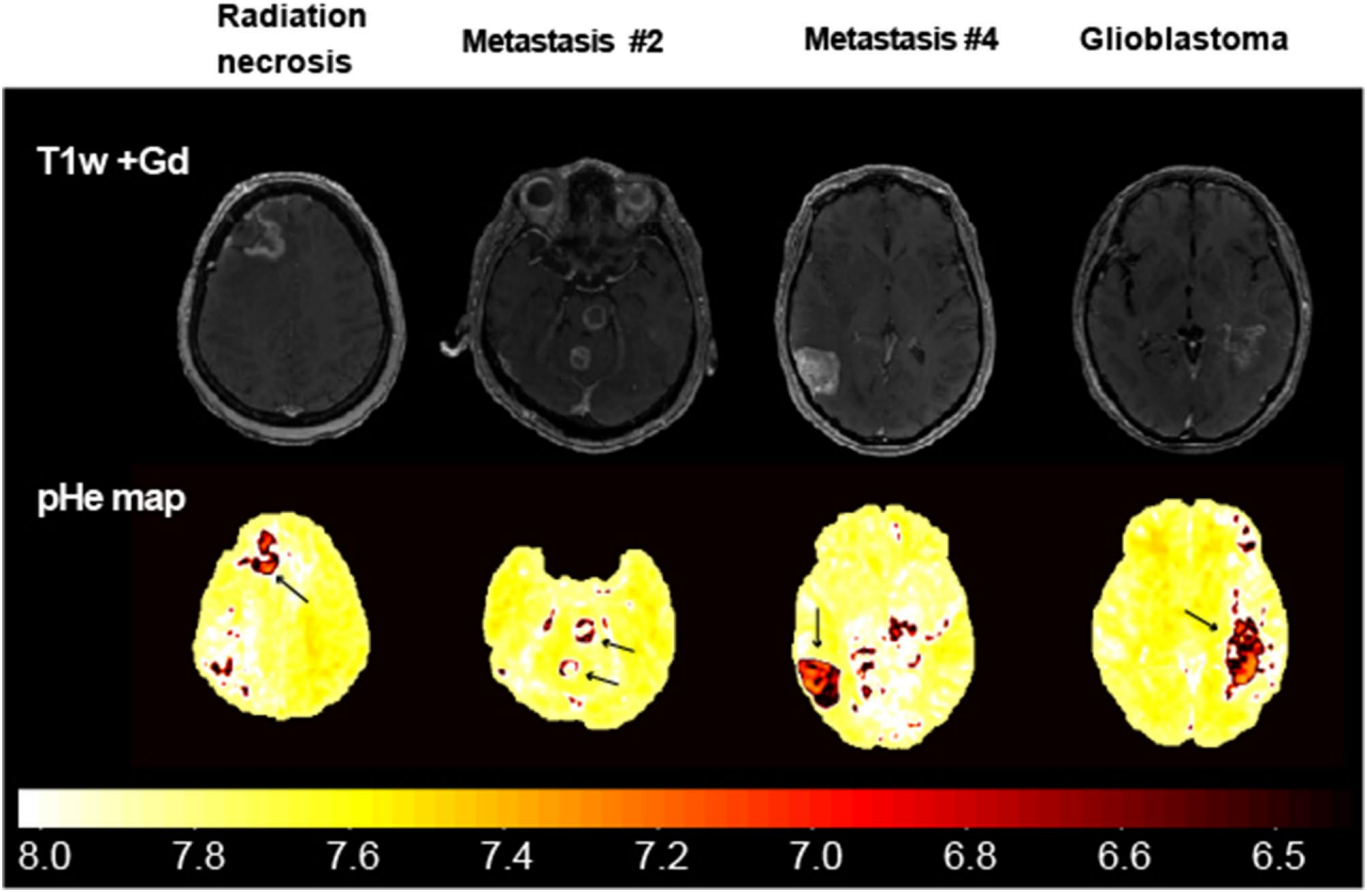
Contrast-enhanced synthetic T1w image-derived QPM and pHe maps. The synthetic T1w and pHe maps indicate contrast-enhanced lesions and pHe changes with a color scale bar [pH 6.5–8.0].

The primary brain tumor group showed significantly higher mean r1 values than other brain disease groups (P < 0.001). Moreover, the mean r1 of the metastasis group was significantly increased compared with radiation necrosis (P < 0.001). The mean pHe value showed a trend for tumor malignancy having a lower pHe value and primary brain tumor having a significantly lower pHe than other brain diseases (P < 0.001). Moreover, the mean pHe value of the metastasis group was significantly decreased compared with that of the radiation necrosis, indicating that pHe can help evaluate therapeutic efficacy (P < 0.001). Moreover, the synthetic *T*_1_w post contrast administration derived from QPM and pHe maps is shown in Fig. 6. Mean pHe value of 7.49 ± 0.019 was observed in the superior sagittal sinus. This value approximately matched the theoretical blood pH of 7.40.

## Discussion

r1-based CAs have an important potential application in pHe measurement. In the early days, the r1 values were reported to show an increasing/decreasing behavior depending on the pH^14^. This behavior is based on protic exchange and second hydration sphere dynamics that can provide the relaxivity change in acidic environments; therefore, the acidic environment plays a role in modulating relaxation changes^13,18,19^. We first performed a phantom study to determine the pH sensitivity of a conventional CA, Gd-BTDO3A. In this experiment, a range of 6.95–7.3 was considered as the pH-sensitive range for the calibration curve, resulting in four parameters: kPa = 6.70, r1base = 3.50, r1acid = 6.50 and n = 1.34. The relaxivity value depends on temperature because the relaxation time of T1 changes linearly with the temperature^20^. In this study, the relationship between relaxivity and pH at room temperature and body temperature (Fig. 3) supports the notion of temperature dependence of relaxivity.

**Figure 7:**
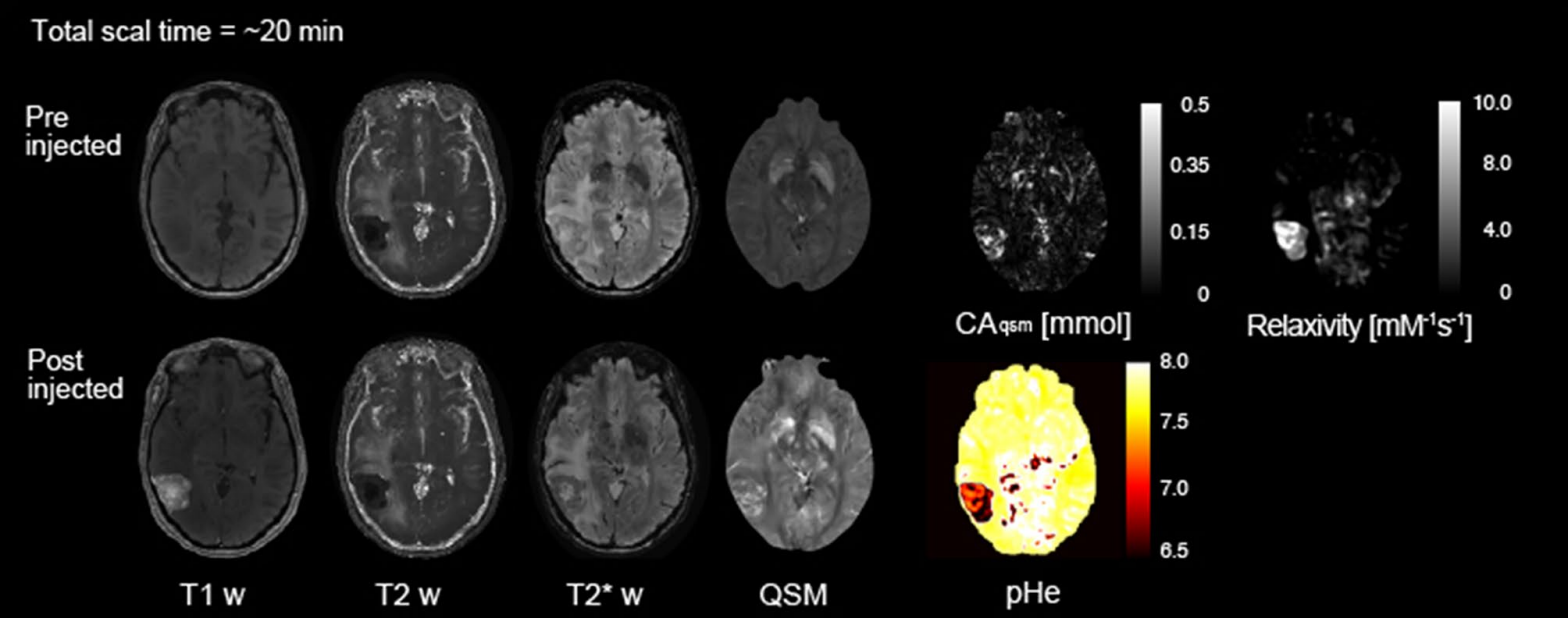
Brain disease evaluation using QPM before and after contrast agent injection. Simultaneous 3D mapping of PD, T1, T2* and susceptibility map can obtain contrast images. Moreover, our method can estimate CA concentration, r1 and pHe mapping by using CA.

In our approach to separately quantify CA concentration and r1, we used QPM because it can simultaneously measure the quantitative maps of both relaxation and susceptibility, leading to a clinically feasible acquisition time (~ 20 min). We then measured QPM in patients before and after administering Gd-BTDO3A and separately quantified the CA concentration and r1 from *R*_1 subtraction_ and CA_qsm_ maps. Both maps had a high correlation with each brain disease (see Fig. 4). This means that *R*_1 subtraction_ and CA_qsm_ maps can separately detect the Gd3+ relaxation change in tumor lesions. Thus, QPM has an important practical advantage for detecting Gd3+ behavior on clinical scan time. An additional advantage of QPM is that standard T1w, T2w, T2*w, fluid-attenuated inversion recovery (FLAIR) images can be simultaneously obtained both before and after CA injection, as shown in Fig. 7, which displays actual post-processed synthetic MRI data of a patient with metastasis #4. According to the present results, using QPM to determine T1w, T2w, T2*w, FLAIR, and pHe estimates would lead to more accurate characterization and identification of brain lesions.

A pHe map was then obtained by applying the result of the phantom experiment to the r1 map, showing that the mean pHe value decreased in the tumor grade group, as shown in Fig. 5. This observation is consistent with that of earlier studies showing that the average pHe reaches approximately 6.8–7.2 in solid tumors because of the Warburg effect^21–23^. Additionally, the blood pHe was measured on the superior sagittal sinus to investigate whether the blood pH can be correctly estimated. As a result, the mean value of 7.49 ± 0.019 was observed. This value approximately matched the theoretical blood pH of 7.40, our approach can approximately quantify pHe, if relaxivity values are not high.

Our study proposes a new method using QPM to evaluate r1 and CA concentration in contrast-enhanced lesions, showing the possibility of evaluating tumors’ pathophysiological changes. Furthermore, r1 values may be used as pHe markers. These indices may be useful for brain tumor management.

**Figure 8:**
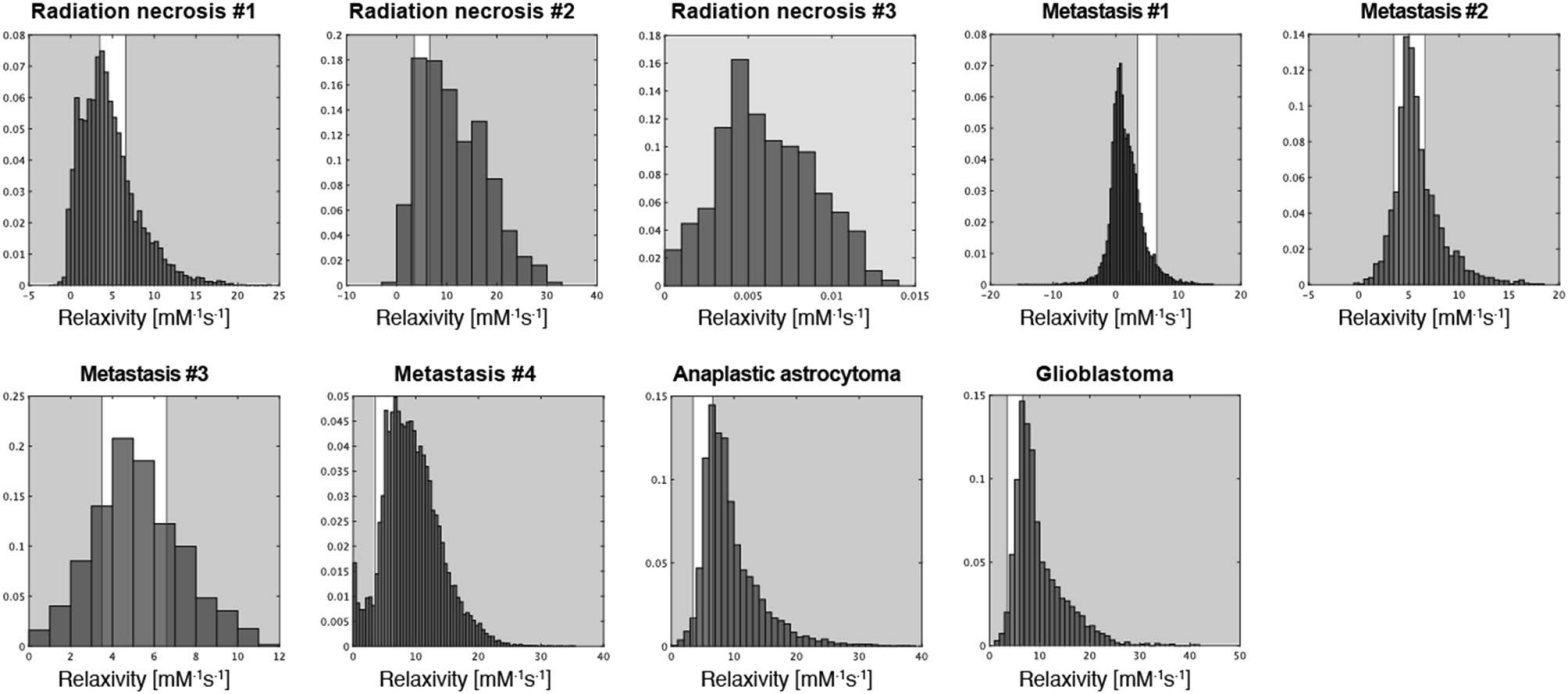
Histogram of relaxivity in the tumor lesion for each patient. White and gray areas indicate pH sensitive and insensitive ranges against the relaxivity distribution, respectively.

A major limitation of the conventional CA based pHe measurement is that sensitivity to CA is limited in a narrow pH range in clinical measurements. In this study, a pHe-relaxivity calibration curve was obtained with four parameters: kPa = 6.70, r1_base_ = 3.50, r1_acid_ = 6.50 and n = 1.34, indicating a sensitivity range from 3.50 to 6.50. Figure 8 shows a histogram of the relaxivity in the tumor lesion for each patient. As a result, a narrow sensitive range (white area in Fig. 8) was observed against the relaxivity distribution, which is in agreement with the peak of the relaxivity distribution except for radiation necrosis #3 and Metastasis #1. Additionally, we believe our results to be acceptable because the mean pHe value significantly decreased in subjects with primary tumors compared to that in those with radiation necrosis. In future work, a newly developed pH-sensitive contrast agent may be required to increase the range and accuracy of pH sensitivity. Although TmDOTP5 has been studied as a pH sensitive CA, it is not approved for clinical use^24–26^. Another major limitation is that despite showing the pH-relaxivity curve, the accuracy of pH obtained using our methodology has not been validated in detail. Further in vitro studies using QPM are needed to determine the pH accuracy. QPM was optimized to achieve adequate relaxation time and susceptibility mapping of brain tissue. Therefore, at present, simultaneous imaging of both T1 and susceptibility mapping are difficult in a phantom experiment.

Our pHe measurement is specifically designed for the acidic extracellular space of solid tumors. However, our method is not restricted to brain tissue computation and can be adapted to other tissues as data acquisition is based on 3D spoiled gradient echo pulse sequences. Therefore, our methods could assist in the future application of pHe mapping to other tissues.

In conclusion, this study demonstrated that QPM can separately quantify r1 and CA concentration in brain tumors and that pHe mapping of brain tumors could serve as a biomarker for tumor characterization. In particular, our method has clinical potential for assessing the treatment effects.

## Data Availability

The authors confirm that the data supporting the findings of this study are available within the article.
